# A Phase 2A Trial of the Safety and Tolerability of Increased Dose Rifampicin and Adjunctive Linezolid, With or Without Aspirin, for Human Immunodeficiency Virus–Associated Tuberculous Meningitis: The LASER-TBM Trial

**DOI:** 10.1093/cid/ciac932

**Published:** 2022-12-09

**Authors:** Angharad G Davis, Sean Wasserman, Cari Stek, Mpumi Maxebengula, C Jason Liang, Stephani Stegmann, Sonya Koekemoer, Amanda Jackson, Yakub Kadernani, Marise Bremer, Remy Daroowala, Saalikha Aziz, Rene Goliath, Louise Lai Sai, Thandi Sihoyiya, Paolo Denti, Rachel P J Lai, Thomas Crede, Jonathan Naude, Patryk Szymanski, Yakoob Vallie, Ismail Abbas Banderker, Muhammed S Moosa, Peter Raubenheimer, Sally Candy, Curtis Offiah, Gerda Wahl, Isak Vorster, Gary Maartens, John Black, Graeme Meintjes, Robert J Wilkinson

**Affiliations:** Francis Crick Institute, London, United Kingdom; Faculty of Life Sciences, University College London, London, United Kingdom; Wellcome Centre for Infectious Diseases Research in Africa, Institute of Infectious Disease and Molecular Medicine, University of Cape Town, Observatory, Republic of South Africa; Wellcome Centre for Infectious Diseases Research in Africa, Institute of Infectious Disease and Molecular Medicine, University of Cape Town, Observatory, Republic of South Africa; Department of Medicine, University of Cape Town, Observatory, Republic of South Africa; Wellcome Centre for Infectious Diseases Research in Africa, Institute of Infectious Disease and Molecular Medicine, University of Cape Town, Observatory, Republic of South Africa; Department of Infectious Diseases, Imperial College London, London, United Kingdom; Wellcome Centre for Infectious Diseases Research in Africa, Institute of Infectious Disease and Molecular Medicine, University of Cape Town, Observatory, Republic of South Africa; Biostatistics Research Branch, National Institute of Allergy and Infectious Diseases, Bethesda, Maryland, USA; Wellcome Centre for Infectious Diseases Research in Africa, Institute of Infectious Disease and Molecular Medicine, University of Cape Town, Observatory, Republic of South Africa; Wellcome Centre for Infectious Diseases Research in Africa, Institute of Infectious Disease and Molecular Medicine, University of Cape Town, Observatory, Republic of South Africa; Wellcome Centre for Infectious Diseases Research in Africa, Institute of Infectious Disease and Molecular Medicine, University of Cape Town, Observatory, Republic of South Africa; Wellcome Centre for Infectious Diseases Research in Africa, Institute of Infectious Disease and Molecular Medicine, University of Cape Town, Observatory, Republic of South Africa; Wellcome Centre for Infectious Diseases Research in Africa, Institute of Infectious Disease and Molecular Medicine, University of Cape Town, Observatory, Republic of South Africa; Wellcome Centre for Infectious Diseases Research in Africa, Institute of Infectious Disease and Molecular Medicine, University of Cape Town, Observatory, Republic of South Africa; Department of Infectious Diseases, Imperial College London, London, United Kingdom; Wellcome Centre for Infectious Diseases Research in Africa, Institute of Infectious Disease and Molecular Medicine, University of Cape Town, Observatory, Republic of South Africa; Wellcome Centre for Infectious Diseases Research in Africa, Institute of Infectious Disease and Molecular Medicine, University of Cape Town, Observatory, Republic of South Africa; Wellcome Centre for Infectious Diseases Research in Africa, Institute of Infectious Disease and Molecular Medicine, University of Cape Town, Observatory, Republic of South Africa; Wellcome Centre for Infectious Diseases Research in Africa, Institute of Infectious Disease and Molecular Medicine, University of Cape Town, Observatory, Republic of South Africa; Division of Clinical Pharmacology, Department of Medicine, University of Cape Town, Observatory, Republic of South Africa; Francis Crick Institute, London, United Kingdom; Department of Infectious Diseases, Imperial College London, London, United Kingdom; Department of Medicine, Mitchells Plain Hospital, Cape Town, South Africa; Department of Medicine, Mitchells Plain Hospital, Cape Town, South Africa; Department of Medicine, Mitchells Plain Hospital, Cape Town, South Africa; Department of Medicine, New Somerset Hospital, Cape Town, South Africa; Department of Medicine, Mitchells Plain Hospital, Cape Town, South Africa; Department of Medicine, New Somerset Hospital, Cape Town, South Africa; Department of Medicine, University of Cape Town, Observatory, Republic of South Africa; Division of Diagnostic Radiology, University of Cape Town, Groote Schuur Hospital, Observatory, Republic of South Africa; Department of Neuroradiology, Imaging Department, Royal London Hospital, Barts Health NHS Trust, London, United Kingdom; Department of Medicine, Walter Sisulu University, Mthatha, Republic of South Africa; Division of Diagnostic Radiology, University of Cape Town, Groote Schuur Hospital, Observatory, Republic of South Africa; Wellcome Centre for Infectious Diseases Research in Africa, Institute of Infectious Disease and Molecular Medicine, University of Cape Town, Observatory, Republic of South Africa; Division of Clinical Pharmacology, Department of Medicine, University of Cape Town, Observatory, Republic of South Africa; Department of Medicine, Walter Sisulu University, Mthatha, Republic of South Africa; Wellcome Centre for Infectious Diseases Research in Africa, Institute of Infectious Disease and Molecular Medicine, University of Cape Town, Observatory, Republic of South Africa; Department of Medicine, University of Cape Town, Observatory, Republic of South Africa; Francis Crick Institute, London, United Kingdom; Faculty of Life Sciences, University College London, London, United Kingdom; Wellcome Centre for Infectious Diseases Research in Africa, Institute of Infectious Disease and Molecular Medicine, University of Cape Town, Observatory, Republic of South Africa; Department of Medicine, University of Cape Town, Observatory, Republic of South Africa; Department of Infectious Diseases, Imperial College London, London, United Kingdom

**Keywords:** tuberculous meningitis, HIV, rifampicin, linezolid, aspirin

## Abstract

**Background:**

Drug regimens that include intensified antibiotics alongside effective anti-inflammatory therapies may improve outcomes in tuberculous meningitis (TBM). Safety data on their use in combination and in the context of human immunodeficiency virus (HIV) are needed to inform clinical trial design.

**Methods:**

We conducted a phase 2, open-label, parallel-design, randomized, controlled trial to assess the safety of high-dose rifampicin, linezolid, and high-dose aspirin in HIV-associated TBM. Participants were randomized (1.4:1:1) to 3 treatment arms (1, standard of care [SOC]; 2, SOC + additional rifampicin [up to 35 mg/kg/d] + linezolid 1200 mg/d reducing after 28 days to 600 mg/d; 3, as per arm 2 + aspirin 1000 mg/d) for 56 days, when the primary outcome of adverse events of special interest (AESI) or death was assessed.

**Results:**

A total of 52 participants with HIV-associated TBM were randomized; 59% had mild disease (British Medical Research Council (MRC) grade 1) vs 39% (grade 2) vs 2% (grade 3). AESI or death occurred in 10 of 16 (63%; arm 3) vs 4 of 14 (29%; arm 2) vs 6 of 20 (30%; arm 1; *P* = .083). The cumulative proportion of AESI or death (Kaplan–Meier) demonstrated worse outcomes in arm 3 vs arm 1 (*P* = .04); however, only 1 event in arm 3 was attributable to aspirin and was mild. There was no difference in efficacy (modified Rankin scale) between arms.

**Conclusions:**

High-dose rifampicin and adjunctive linezolid can safely be added to the standard of care in HIV-associated TBM. Larger studies are required to determine whether potential toxicity associated with these interventions, particularly high-dose aspirin, is outweighed by mortality or morbidity benefit.

**Clinical Trials Registration:**

NCT03927313.

## Teaser

In this phase 2A, randomized, controlled trial, we demonstrate that high-dose rifampicin and adjunctive linezolid is safe in adult human immunodeficiency virus–associated tuberculous meningitis. Larger studies are required to evaluate potential toxicity with high-dose aspirin in relation to benefit on morbidity and mortality.

Tuberculous meningitis (TBM) is the most severe form of tuberculosis (TB). With currently available treatment, mortality is high, up to 50% in those coinfected with human immunodeficiency virus (HIV) [1]. In those who survive, there is a high burden of disability due to neurological sequelae such as stroke [2], epilepsy [3], inflammatory complications within the spinal cord [4], and cognitive impairment [5]. Drug regimens used to treat TBM are largely based on those used in pulmonary TB. There is a need to design and evaluate new regimens that account for the differing ability of drugs to penetrate the central nervous system (CNS) while simultaneously counteracting the dysregulated immune response that occurs in TBM in order to improve outcomes for this disease.

Rifampicin at standard adult doses seldom achieves adequate cerebrospinal fluid (CSF) concentrations [6]; it is therefore likely that higher doses would increase CNS bactericidal activity. Two randomized, controlled trials (RCTs) in TBM have evaluated high-dose rifampicin (13 mg/kg intravenous [IV] [7] and 15 mg/kg oral [8]) with conflicting results. A recent pharmacokinetic (PK) study suggested that doses higher than 15 mg/kg may improve outcomes by demonstrating approximately 8-fold and approximately 6-fold higher CSF exposures with 35 mg/kg (oral) and 20 mg/kg (IV) doses, respectively, compared with standard oral dose (10 mg/kg) [9]. Linezolid, now part of the World Health Organization recommended treatment in drug-resistant TB [10], is known to have broad tissue penetration, including into the CNS [11]. Two observational studies of linezolid found favorable clinical and laboratory outcomes in TBM [12, 13]. However, frequently reported hematological and neuropathic toxicity are concerning [14]. In TBM, this toxicity, which is rarely severe [15] and largely reversible [16], may be acceptable. Aspirin targets key pathogenic processes that occur during TBM: inhibition of thromboxane and platelet aggregation at lower doses (75 mg daily) [17] and inhibition of proinflammatory prostaglandins and thromboxane A_2_ at higher doses (>600 mg daily) [18]. The latter may be further augmented by aspirin-related production of pro-resolving lipid mediators [19]. Three RCTs have evaluated aspirin at varying doses (75 mg daily to 1000 mg/d) with varying outcomes in TBM [20–22]. The latest of these demonstrated reduction in infarcts and death with 1000 mg of aspirin compared with placebo in patients without HIV with microbiologically confirmed TBM [20]. The safety of high-dose aspirin, which may lead to anti-inflammatory effects, has yet to be evaluated in the context of HIV and in conjunction with adjunctive antimicrobial drugs.

A number of clinical trials are investigating either the PK properties of linezolid (NCT04021121, NCT03537495) or efficacy of high-dose rifampicin (ISRCTN15668391) as single adjunctive therapies in TBM, and 1 phase 3 trial is evaluating the efficacy of linezolid, high-dose rifampicin, and lower-dose aspirin (NCT04145258). However, safety data on the use of rifampicin and linezolid in combination, of high-dose aspirin in combination with intensified antibiotics, and in the context of HIV coinfection are absent. LASER-TBM (Linezolid, Aspirin and Enhanced Dose Rifampicin in HIV-TBM) aimed to generate much-needed safety data on the use of enhanced antimicrobial therapy including higher-dose rifampicin (35 mg/kg) and linezolid (1200 mg reducing to 600 mg daily) with or without adjunctive high-dose aspirin (1000 mg daily) in HIV-associated TBM to inform their use in definitive clinical trials.

## METHODS

LASER-TBM was an open-label, parallel-group, randomized, multiarm, phase 2A trial; participants were randomized to 1 of 3 treatment arms (described below). Investigational drugs were given for the first 56 days of therapy. Primary end point data were collected at day 56. Treatment was subsequently continued per South African national guidance. Participants completed study follow-up at 6 months. Interim analysis for safety was performed by an independent data and safety monitoring board (DSMB) after every 15 participants enrolled. A full version of the study protocol is published elsewhere [23].

### Study Participants and Sites

Adults aged ≥18 years with a diagnosis of possible, probable, or definite TBM [24] and confirmed HIV-1 seropositivity were eligible for enrollment. Exclusion criteria are listed in Supplementary Box 1. Written informed consent was obtained from participants; in those without capacity to consent, proxy consent from next of kin was obtained. In the latter cases, deferred consent was obtained from the patient as soon as they were able. Potential participants were referred while inpatients at 4 government hospitals across South Africa. Subsequent follow-up occurred in inpatient wards and outpatient clinics at respective sites or at 2 TB hospitals in Cape Town.

### Intervention

Participants were randomized to 1 of 3 treatment arms (1.4:1:1). Proportionally more participants were randomized to arm 1 to account for anticipated higher mortality with standard of care compared with the intervention arms. Treatment arms were as follows: arm 1 (standard of care): rifampicin 10 mg/kg, isoniazid (H) 5 mg/kg, ethambutol (E) 15 mg/kg, and pyrazinamide (Z) 25 mg/kg daily for 56 days; arm 2: as per arm 1 plus adjunctive 25 mg/kg rifampicin (total dose, 35 mg/kg) and linezolid (1200 mg for 28 days, reducing to 600 mg for 28 days) daily for 56 days; and arm 3: as per arm 2 plus adjunctive aspirin (1000 mg) daily for 56 days.

Dosing was calculated by weight bands, published elsewhere [23]. After 56 days, participants were referred to government TB facilities to receive continuation therapy (rifampicin, 10 mg/kg/d and isoniazid, 5 mg/kg/d) for 7 months per national guidelines [25].

Participants allocated to arms 2 and 3 were further randomized (1:1) to receive oral rifampicin 35 mg/kg or IV rifampicin 20 mg/kg daily for the first 3 days of therapy (in addition to HZE and linezolid ± aspirin, according to the experimental arm). Results of this PK substudy are published elsewhere [26].

All participants in the LASER-TBM trial not on antiretroviral therapy (ART) were referred to local government clinics to initiate ART according to the Western Cape Consolidated Guidelines for HIV Treatment. Given the potential interaction between rifampicin and lopinavir/ritonavir and between dolutegravir, clinics were advised to double doses of these drugs until 2 weeks after anti-TB treatment had ceased.

### Outcome Measures

The primary end point of the study was the cumulative proportion of participants experiencing adverse events of special interest (AESI) or dying by 56 days. AESI were selected based on anticipated toxicity related to the 2 interventional arms: bleeding (gastrointestinal and intracerebral hemorrhage), hematological (anemia, neutropenia, thrombocytopenia), transaminitis, and neuropathic (peripheral and optic neuropathy; Table 1). Secondary end points are listed in Box 1.

Box 1: **Secondary End Points**

- Death and severe disability by 56 days
- Death by 56 days and 180 days
- Disability at 56 days and 180 days
- Incidence of grade 3 or 4 adverse events
- Permanent discontinuation of study drugs
- Severity and frequency of hematological and neurological adverse events of special interest related to linezolid use
- Severity and frequency of major bleeding (gastrointestinal and intracerebral) related to aspirin use
- Occurrence of tuberculous meningitis immune reconstitution inflammatory syndrome (IRIS) assessed using the modified International Network for the Study of HIV-associated IRIS criteria [27]
- Magnetic resonance imaging and computed tomography changes at day 56

**Table 1.** Adverse Events of Special Interest Assessed in the Trial

Adverse Event of Special Interest	Investigational Product	Objective Measure
Gastrointestinal hemorrhage	Aspirin	Clinical and laboratory measures to suggest hemorrhage
Intracerebral hemorrhage	Aspirin	Radiological evidence of hemorrhage
Transaminitis	Rifampicin	Alanine transaminase, bilirubin (DAIDS criteria, grades 3 and 4)
Anemia	Linezolid	Hemoglobin (DAIDS criteria, grades 3 and 4)
Neutropenia	Linezolid	Neutrophils (DAIDS criteria, grades 3 and 4)
Thrombocytopenia	Linezolid	Platelet count (DAIDS criteria, grades 3 and 4)
Peripheral neuropathy	Linezolid	1 grade increase on the brief peripheral neuropathy score and/or a 2 grade change in any modality on the modified total neuropathy score
Change in logarithm of the minimum angle of resolution score (visual acuity)	Linezolid	Change of 0.2 on the logarithm of the minimum angle of resolution/tumbling E chart

Abbreviation: DAIDS, Division of AIDS.

### Study Schedule and Safety Outcome Measures

Participants were screened and enrolled within 5 days of commencing TB treatment. They were assessed at 5 subsequent study visits (days 3, 7, 14, 28, and 56) at which point they were referred to government TB clinics for continuation therapy (Supplementary Figure 1). Participants were assessed clinically at 1 additional time point (day 180), in person or telephonically. Where feasible, imaging (computed tomography and/or magnetic resonance imaging) was performed at baseline and day 56. A neurological assessment that included full motor/sensory and cranial nerve examination was performed at each study visit. Participants’ functional status was assessed using the modified Rankin scale (MRS) at each study visit up to day 56 and telephonically at day 180. Assessment for AESI was also made at each study visit up to and including day 56 involving a clinical history to assess symptoms of bleeding (gastrointestinal or intracerebral), brief peripheral neuropathy score, and modified total neuropathy score to assess for peripheral neuropathy, a logarithm of the minimum angle of resolution (LogMAR)/tumbling E chart to assess for optic neuropathy, a full blood count to assess for hematological abnormalities, and liver function tests for transaminitis. A list of clinical outcome measures and assessments performed at each study visit are listed in the published protocol [23].

### Study Oversight

An independent DSMB oversaw the safety of the trial and advised to continue recruitment without change after review of primary end point data for each 15 participants enrolled.

Approval for the trial was granted by the University of Cape Town Human Research Ethics Committee, Walter Sisulu University Human Research Committee, and the South African Health Products Regulatory Authority. The trial was registered on the South African National Clinical Trials Register, Pan African National Clinical Trials Register, and clinicaltrials.gov.

## Statistical Considerations

### Sample Size

No formal statistical power calculation was performed. Even as single adjunctive therapies, there were limited available data on the use of these drugs in TBM to predict the likely rate of AESI and/or death. Given this would be further complicated when considering likely event rate when combined, it was felt that it would be more pragmatic to create a recruitment target of 100 participants with frequent blinded review of cumulative safety events by an independent DSMB. A secondary aim of LASER-TBM was to serve as a planning study to generate PK and safety data to inform a phase 3 RCT of intensified treatment in TBM (NCT04145258), which in part would influence resulting sample size.

The decision to stop recruitment prior to 100 participants enrolled was due to the rate of recruitment being slower than anticipated due to the coronavirus disease 2019 pandemic; funding for the trial was due to cease in March 2021 and therefore recruitment could take place up until January 2021; and DSMB review of safety data (both during recruitment to the trial and following enrollment of last recruit) had revealed no reason for the planned RCT to not go ahead.

### Statistical Analyses

Analysis was performed in GraphPad Prismv.9.0 and Rv.3.6.0. The primary analysis was performed in the modified intention-to-treat population (received any dose of the study drug). A sensitivity analysis was planned for the per-protocol population (completed treatment as specified in the protocol). However, given the small sample size and since these populations were similar, here we report the most conservative analysis (modified intention-to-treat).

The primary end point, frequency of AESI or death (data censored at the first event prior to day 56), was summarized and compared across arms using a χ2 test. A time-to-event analysis was performed for worst grade (in each individual participant) AESI or death; comparisons between study groups were made using the log-rank test. Neurological disability (MRS) and radiological outcomes at day 56 were compared across arms using a *χ*^2^ test. We used spaghetti plots to visually represent longitudinal CSF parameters (lymphocytes, polymorphonuclear cells, protein, and glucose) over time and *t* tests to compare longitudinal summaries (mean and standard deviation) of each individual trajectory across treatment arms.

Details of further analysis can be found in the published statistical analysis plan [23].

## RESULTS

**Figure 1.**
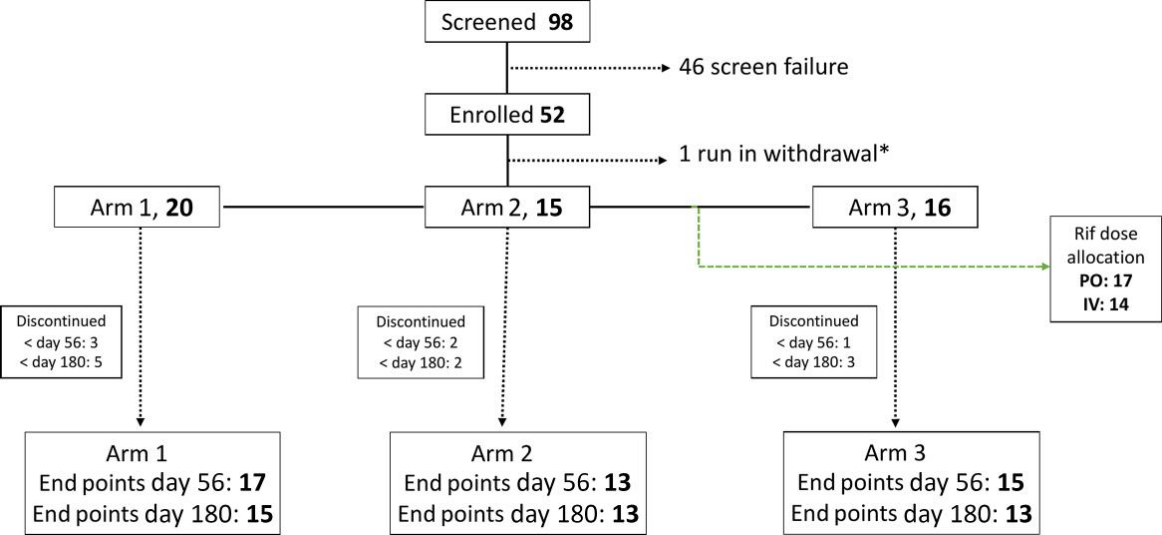
Consolidated Standards of Reporting Trials diagram describing recruitment and arm allocation. One participant was randomized but excluded prior to any study investigational product (IP) being dispensed due to emergence of an exclusion criterion (estimated glomerular filtration rate <20) on a hospital blood test performed prior to randomization. Another participant was excluded from the modified intention-to-treat analysis as they died prior to receiving any dose of study drug. Six participants discontinued the study prior to day 56, and an additional 4 participants discontinued between day 56 and day 180 (Supplementary Table 2). Reasons for screening exclusions and early study withdrawals are listed in Supplementary Tables 1 and 2. *Patient randomized but withdrawn prior to receiving study IP due to emergence of exclusion criteria. Abbreviations: IV, intravenous; PO, by mouth; Rif, rifampicin.

A total of 98 patients were screened and 52 were randomized between June 2019 and January 2021 (Figure 1). Reasons for screening exclusion are summarized in Supplementary Table 1. The baseline characteristics of the participants stratified by treatment arm are described in Table 2.

**Table 2.** Baseline Characteristics

Characteristic	Arm 1 (n = 20)	Arm 2 (n = 15)	Arm 3 (n = 16)
Age, median (IQR), y	39.5 (34–48.5)	37 (34.5–42.5)	41.5 (31.8–46)
Male gender, n (%)	10 (50)	10 (66.7)	16 (62.5)
Uniform case definition, n (%)			
ȃDefinite	8 (40)	3 (20)	6 (37.5)
ȃProbable	5 (25)	4 (26.7)	4 (25.0)
ȃPossible	7 (35)	8 (53.3)	6 (37.5)
British Medical Research tuberculous meningitis grade, n (%)			
ȃ1	11 (55)	11 (73.3)	8 (50)
ȃ2	8 (40)	4 (26.7)	8 (50)
ȃ3	1 (5)	0 (0)	0 (0)
CD4 T-cell count, median (IQR), cells/μL	116.5 (58.6–283)	131 (82.5–186)	158.5 (85.5–331.5)
Human immunodeficiency virus viral load, median (IQR), copies/mL	89 150 (1000–203 711)	37 960 (2428–394 839)	2686 (1361–777 620)
ART status, n (%)			
ȃOn	6 (30)	5 (33)	5 (31)
ȃPrevious^a^	3 (15)	6 (40)	5 (31)
ȃNaive	11 (55)	4 (27)	6 (38)
Duration for those on ART, median (range), wk	288.9 (22.4–459.3)	23.7 (0.4–83.6)	355 (2.9–879.1)
CSF cell count/biochemical data available, n	17	14	13
Polymorphonuclear cells, median (IQR), cells/μL	13 (0–85)	4 (2–16)	16 (3–22)
Lymphocytes, median (IQR), cells/μL	63 (10–259)	79 (11–218)	82 (28–278)
Protein, mg/dL	1.78 (1.13–3.13)	1.89 (0.95–4.2)	1.9 (1.32–2.99)
CSF glucose, mg/dL	2.2 (0.9–2.5)	2.4 (1.9–2.9)	1.7 (1.2–3.3)
Baseline radiology available, n	16	12	11
Hydrocephalus, n (%)	1 (6.3)	1 (8.3)	1 (9.1)
Meningeal enhancement, n (%)	4 (25)	2 (16.7)	6 (54.5)
Tuberculoma(s), n (%)	1 (6.3)	2 (16.7)	2 (18.2)
Infarct(s), n (%)	4 (25)	1 (8.3)	3 (27.3)

Abbreviations: ART, antiretroviral therapy; CSF, cerebrospinal fluid; IQR, interquartile range.

Previous ART refers to participants who at the time of enrollment were not taking ART due to defaulting treatment.

The primary end point analysis was performed in the modified intention-to-treat population (n = 50: arm 1, 20; arm 2, 14; arm 3, 16). The composite primary end point of AESI or death occurred in 6 of 20 participants in arm 1, 4 of 14 in arm 2, and 10 of 16 in arm 3 (*P* = .083). The occurrence of each category of AESI stratified by treatment arm is summarized in Table 3, with further detail on timing and outcome of each of these events listed in Table 4. Frequency of death prior to day 56 was similar across arms (n = 7: arm 1, 3; arm 2, 1; arm 3, 3; *P* = .649) and in no case was cause of death related to study investigational product (Table 5). Grade 3 or 4 adverse events (grade 3: arm 1, 7 vs arm 2, 7 vs arm, 3, 9; *P* = .44 and grade 4: arm 1, 2 vs arm 2, 4 vs arm 3, 4; *P* = .38) or serious adverse events for reasons other than death (arm 1, 6 vs arm 2, 8 vs arm 3, 7; *P* = .37) were similar across treatment arms.

**Table 3.** Adverse Events of Special Interest by Treatment Arm

Adverse Events of Special Interest	Arm 1 (n = 20)	Arm 2 (n = 14)	Arm 3 (n = 16)	*P* Value^a^
Bleeding, n^b^ (%)	0 (0)	0 (0)	1 (6)	.338
Transaminitis, n (%)	0 (0)	0 (0)	2 (13)	.109
Hematological, n (%)	2 (10)	0 (0)	1 (6)	.481
Peripheral neuropathy, n (%)	2 (10)	2 (14)	4 (25)	.46
Change in logarithm of the minimum angle of resolution score, n (%)	0 (0)	2 (14)	2 (13)	.231

Arm 3 vs arm 1.

Individuals with an event.

**Table 4.** Details of Adverse Events of Special Interest by Event

Adverse Event Type	Treatment Arm	Day of Treatment	Division of AIDS Grade	Preexisting	Outcome
Melaena	3	1	1	No	2 episodes of black stool; no associated change in hemoglobin or urea; aspirin stopped and not restarted as per protocol; no further events
Transaminitis	3	16	3	No	Improved to grade 2 but not restarted on high-dose rifampicin at discretion of site PI
Transaminitis	3	6	4	No	Improved, successfully rechallenged with rifafour fixed dose combination; high-dose rifampicin not restarted per protocol.
Neutropenia	1	13	3	Yes	No change in study medication (arm 1)
Neutropenia	3	28	3	No	Linezolid stopped, resolved; not restarted
Anemia	1	28	3	No	No change in study medication (arm 1)
Neurosensory symptoms	1	10	2	No	No change in study medication (arm 1); normal at subsequent visit
Neurosensory symptoms	1	6	2	No	No change in study medication (arm 1); Normal at subsequent visit
Neurosensory symptoms	2	3	1	No	Linezolid stopped; MRI showed anterior cord changes (possible ischemic or inflammatory etiology); not restarted on linezolid, although felt clinically not to be consistent with peripheral neuropathy
Neurosensory symptoms	2	7	1	No	All study medication stopped due to relocation of participant and therefore withdrawal from study; no follow-up BPNS performed
Bilateral lower limb weakness	3	18	2	No	Linezolid stopped; MRI showed changes consistent with tuberculosis radiculopathy/arachnoiditis; linezolid not restarted at discretion of site PI, although clinically unlikely peripheral neuropathy
Paresthesia left leg	3	18	1	No	
Neurosensory symptoms	3	3	1	No	Linezolid stopped; normal at subsequent visit, although linezolid not restarted at discretion of site PI
Neurosensory symptoms	3	10	1	No	Linezolid stopped; participant subsequently died, cause of death not related to linezolid
Asymptomatic increase in brief peripheral neuropathy score	3	13	1	No	Linezolid stopped; normal at subsequent visit; linezolid restarted at 600 mg per protocol
Increase in LogMAR	2	56	1	No	Noted on day 56 visit, therefore, no change in study medication; no follow-up notes
Increase in LogMAR	2	55	1	No	Optic neuropathy ruled out by ophthalmology; linezolid restarted
Change in visual acuity with related change in LogMAR	3	42	4	No	Seen by ophthalmology; diagnosis: parietal stroke ± ethambutol-related optic neuropathy
Increase in LogMAR	3	55	1	No	Noted on day 56 visit, therefore, no change in study medication; no follow-up notes

Abbreviations: LogMAR, logarithm of the minimum angle of resolution; MRI, magnetic resonance imaging; PI, principal investigator.

**Table 5.** Timing and Cause of Death Prior to Day 56

Cause of Death	Treatment Arm	Day of Investigational Product
Renal failure	1	11
TBM	1	15
TBM	1	2
TBM	2	8
TBM	2	0^a^
Pulmonary embolism	3	39
TBM	3	6
TBM	3	3

Abbreviation: TBM, tuberculous meningitis.

Death prior to receiving study investigational product and therefore excluded from modified intention-to-treat population analysis.

**Figure 2.**
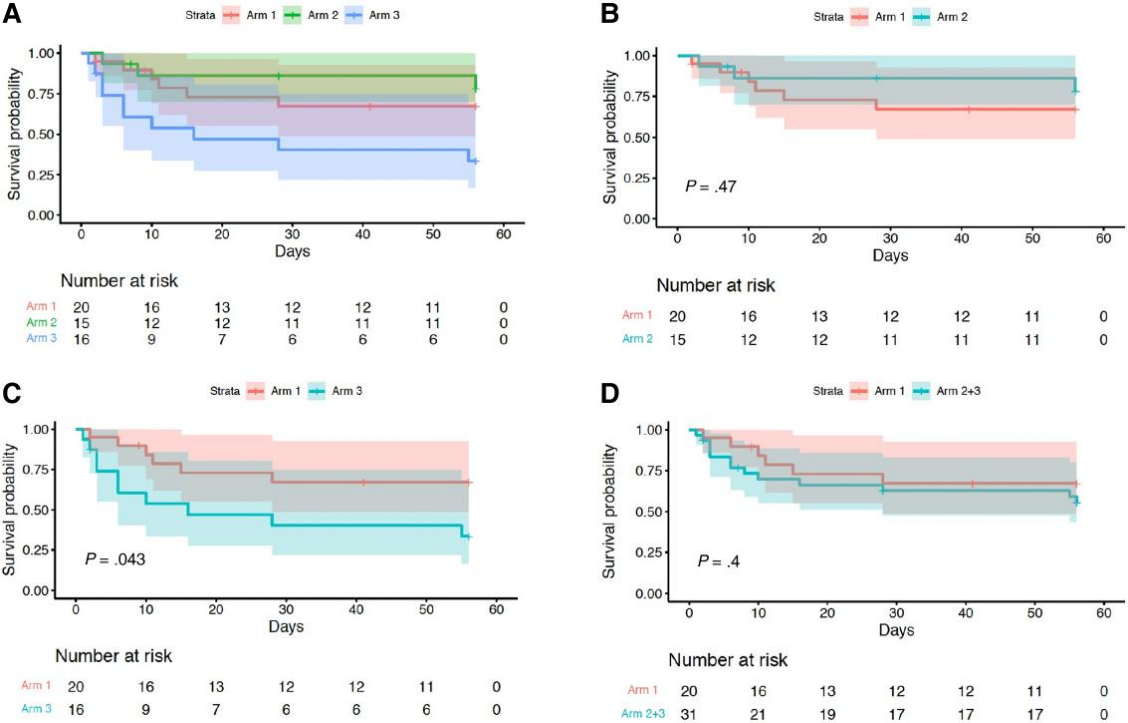
Time to worst-grade adverse events of special interest (AESI) or death. Kaplan–Meier analysis of time to worst grade AESI or death, comparing arms 1, 2, and 3 (*A*), arm 2 vs arm 1 (*B*), arm 3 vs arm 1 (*C*), and arms 2 and 3 combined vs arm 1 (*D*). Arm 3 vs arm 1 (*C*) demonstrated a significant *P* value (*P* = .043); however, all other comparisons showed no significant difference between arms.

**Figure 3.**
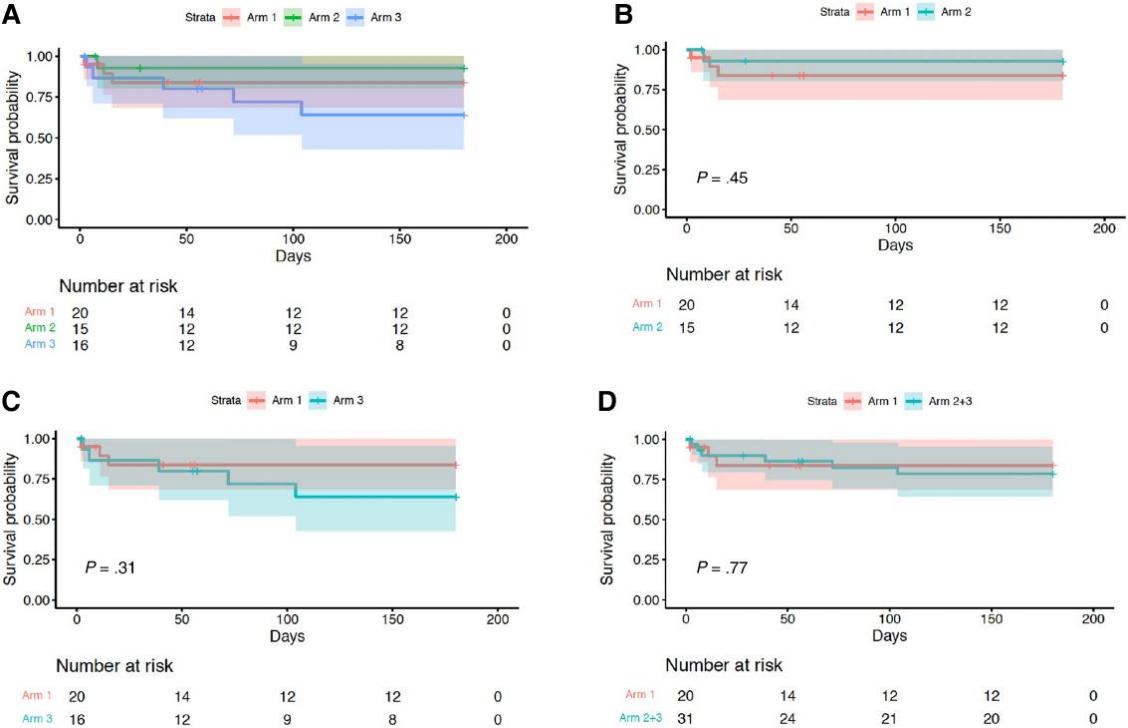
Time to death. Kaplan–Meier analysis of time to death, comparing arms 1, 2, and 3 (*A*), arm 2 vs arm 1 (*B*), arm 3 vs arm 1 (*C*), and arms 2 and 3 combined vs arm 1 (*D*). Note that no analysis demonstrates a statistically significant difference between arms.

**Figure 4.**
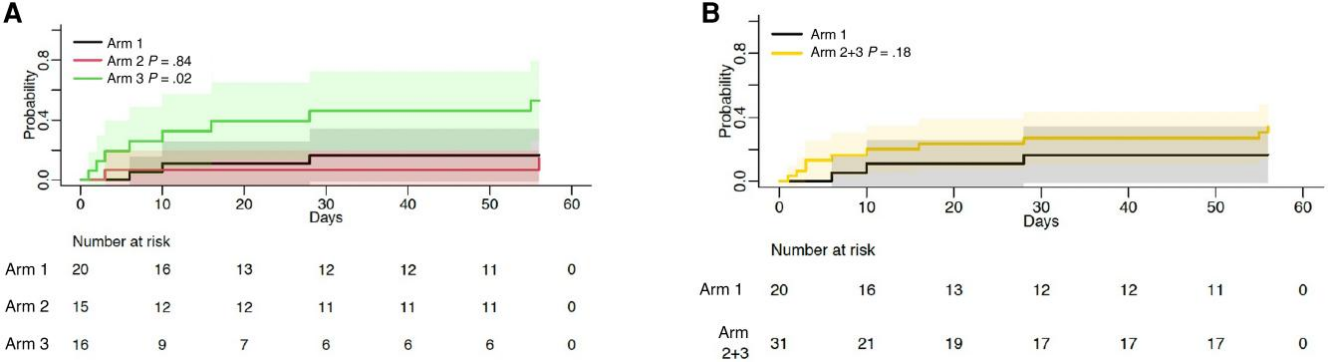
Time to adverse events of special interest (AESI). Kaplan–Meier analysis of time to AESI, comparing arms 1, 2, and 3 (*A*) and arms 2 and 3 combined vs arm 1 (*B*). Note that comparison of time to AESI in arm 3 vs arm 1 demonstrated a statistically significant difference (*P* = .02); however, arm 2 vs arm 1 and arms 2 and 3 combined vs arm 1 were not significantly different (*P* values of .84 and .18, respectively).

The cumulative incidence of the composite end point of worst grade AESI or death at day 56 demonstrated worse outcomes when comparing arm 3 vs arm 1 (*P* = .043), with similar proportions observed in other prespecified analysis (arm 2 vs arm 1, *P* = .3; arm 2 + 3 combined vs arm 1, *P* = .5; Figure 2, log-rank test). Similarly, analysis for death alone demonstrated no difference between arms (Figure 3). The cumulative incidence of AESI was greater in arm 3 vs arm 1 (*P* = .02). However, when arms 2 and 3 were combined and compared with arm 1, this difference was less marked (*P* = .18; Figure 4).

**Figure 5.**
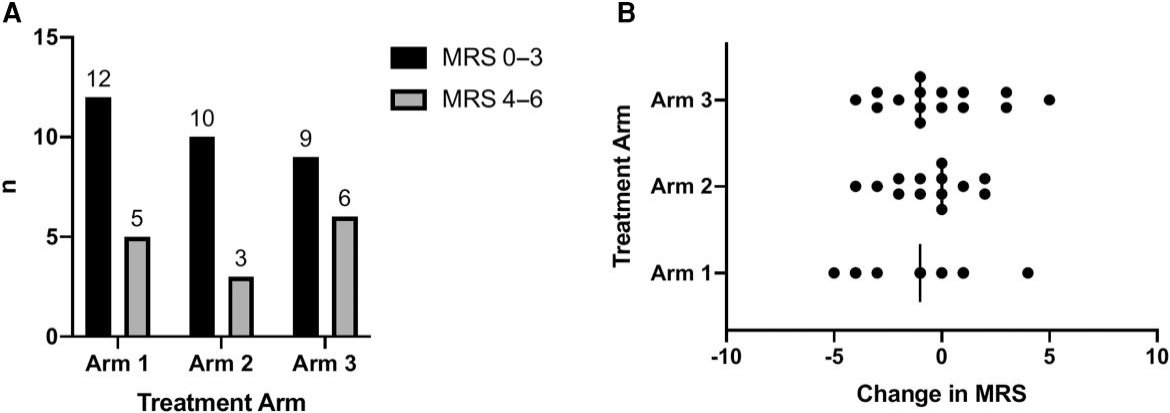
Functional outcome at day 56 as defined by modified Rankin scale (MRS). *A,* Comparison between good outcome (MRS, 0–3) vs bad outcome stratified by arm at day 56 with no significant difference between the 3 treatment arms (*P* = .616). *B,* Change in MRS between enrollment and day 56 across treatment arms with no statistically significant difference between the 3 treatment arms (*P* = .611).

The frequency of grade 5 MRS (severe disability) or death was 4 (arm 1) vs 3 (arm 2) vs 5 (arm 3); *P* = .774. The frequency of good (defined as MRS grade 0–3) and bad outcomes (MRS grade 4–6) was similar across arms (*P* = .616; Figure 5). Post hoc analysis of change in neurological function (as measured by MRS) found similar changes of MRS from baseline to day 56 between the 3 arms (Figure 4*A* ). Neurological sequalae are described in Table 6. Baseline and follow-up imaging was performed in only 9 patients at the time points prespecified within the protocol. Follow-up imaging demonstrated new or worsening leptomeningeal enhancement in 2 of 9 participants (arm 1 and arm 2), new evidence of infarction in 2 of 9 participants (arm 1 and arm 2) and new or worsening tuberculomas in 2 of 9 participants (arm 1 and arm 2), which was associated with worsening sulcal effacement in 1 of 9 participant (arm 1).

**Table 6.** Neurological Sequalae

Neurological Sequalae	Arm 1	Arm 2	Arm 3
	n = 20	n = 15	n = 16
Inflammatory myelopathy	…	1	…
Anterior cord ischemia	…	1	…
Radiculopathy/arachnoiditis	…	…	1
New-onset lower limb weakness^a^	1	…	…
Clinical presentation of stroke	0	2	1
New isolated cranial nerve palsy	0	1	0
New onset seizures	5	2	3
IRIS [27] recorded by day 56 (of which neurological IRIS [28])	2 (1)	2 (2)	3 (2)

Few IRIS events occurred (arm 1, 2; arm 2, 2; arm 3, 3), of which 4 of 7 were defined as neurological IRIS. Within the first 56 days of treatment, 4 participants developed new-onset lower limb weakness (tuberculosis myelopathy, 2; tuberculosis radiculomyelopathy/arachnoiditis, 1; other [no cause found prior to death], 1); 3 participants developed new-onset hemiplegia; 1 patient developed new-onset isolated cranial nerve palsy (lower motor neuron VII). Thirteen participants presented with new-onset seizures at tuberculous meningitis diagnosis. An additional 9 participants had new-onset seizures within the first 2 months of follow-up (arm 1, 5; arm 2; 2; arm 3, 2; *P* = .54).

Abbreviation: IRIS, immune reconstitution inflammatory syndrome.

No cause found prior to death.

**Figure 6.**
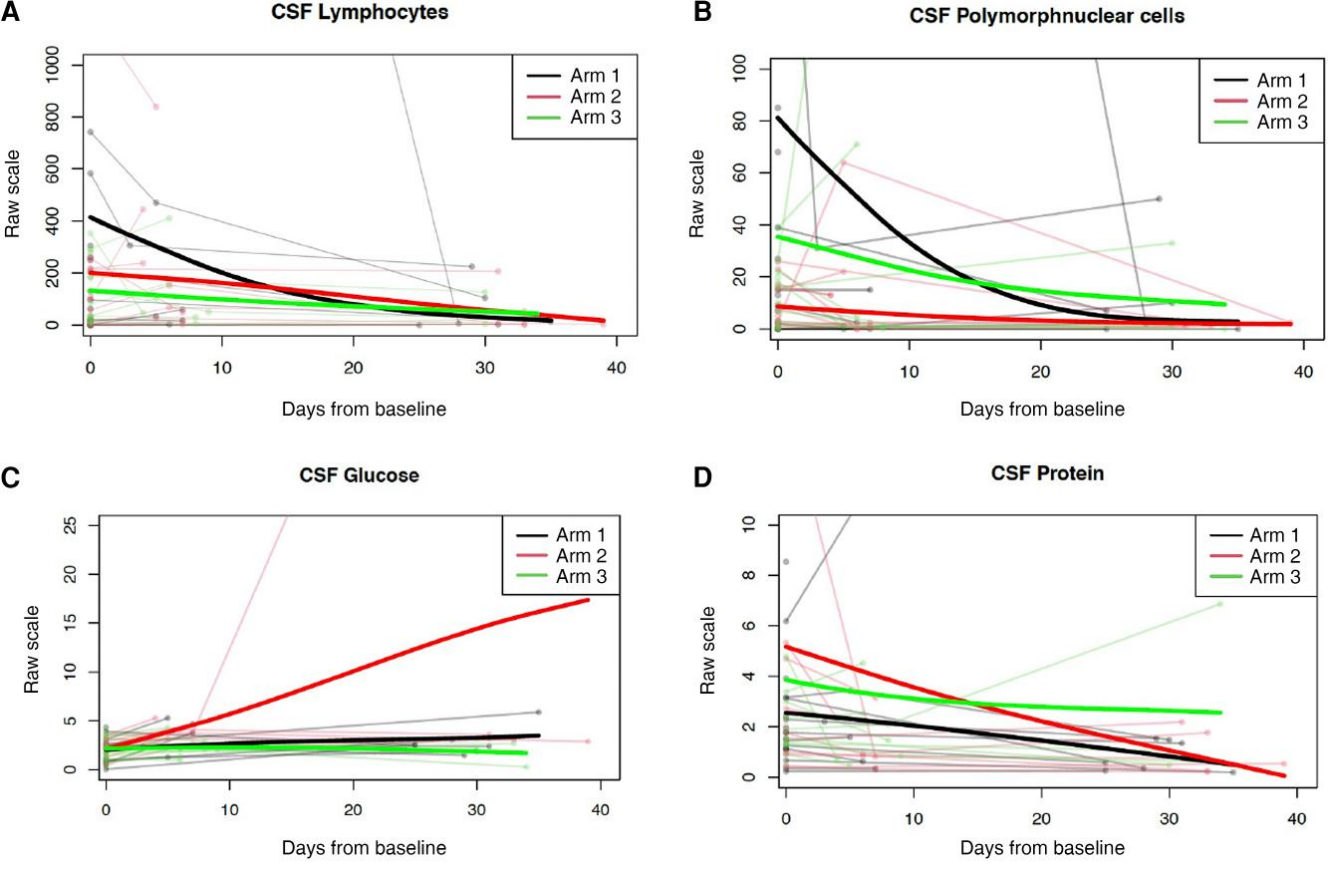
Change in cerebrospinal fluid (CSF) parameters over time. Spaghetti plots for CSF parameters, lymphocyte count (*A*), polymorphonuclear cells (*B*), glucose (*C*), and protein (*D*), plotted as individual values over time (faint lines), with mean values for each treatment arm represented by superimposed line (bold lines). Individual values are plotted, and the superimposed bold line represents the mean values at each time point in each treatment arm as per the color key. The *t* tests comparing mean and variance at each time point demonstrated no difference between arms.

Spaghetti plot analysis of longitudinal CSF parameters over time demonstrated a downward trend of parameters across all 3 treatment arms (Figure 6), not statistically significant between arms.

## DISCUSSION

LASER-TBM was a phase 2A RCT that evaluated the safety of high-dose rifampicin (35 mg/kg daily), adjunctive linezolid, and high-dose aspirin for the first 56 days of treatment in HIV-associated TBM. Primary end point analysis showed no significant difference in the incidence of AESI or death between treatment arms. There was no difference in death or disability at day 56 across arms, and a similar frequency of clinical or radiological events occurred in each arm.

Although secondary analysis revealed a significantly higher number of events (AESI or death) in arm 3 vs arm 1 (*P* = .04), it is reassuring that no deaths were attributed to aspirin. Only 1 bleeding event occurred, after 1 dose of aspirin, and it was resolved immediately following discontinuation and was not associated with any laboratory markers to suggest significant gastrointestinal bleeding. Toxicity attributable to linezolid was similarly mild; of 7 “peripheral neuropathy” events that occurred in participants randomized to experimental arms, 3 of 7 were due to an alternative cause and 2 of 7 had recovered prior to the subsequent study visit. This is perhaps expected given that most recent studies showing a median time to onset of neuropathy after 10 weeks of treatment [15, 16]. Only 1 participant developed a change in visual acuity, which may have been due to linezolid, although on review by an ophthalmologist was assessed to be more likely due to ethambutol. The number of participants in whom potential abnormalities were detected using the LogMAR/tumbling E assessments compared with the confirmed number of cases of optic neuropathy calls into question the specificity of these outcome measures. Given linezolid's potential for treating TBM and drug-resistant TB, better outcome measures are needed to reliably detect abnormalities attributable to these drugs in clinical and research settings in order to prevent overestimation of toxicity. Toxicity due to rifampicin was similarly infrequent with only 2 participants developing clinically significant transaminitis. In both cases, the transaminitis was resolved with treatment interruption. These results suggest that toxicity associated with the enhanced antitubercular regimen (rifampicin 35 mg/kg and adjunctive linezolid) is not common when used in combination for 2 months to treat HIV-associated TBM. This is encouraging in the context of a disease where no specific evidenced-based antitubercular regimen exists and provides rationale for the ongoing phase 3 RCT (NCT04145258) where participants receive both high-dose rifampicin and linezolid at doses identical to those given in this study.

There are limitations to this study. Although no formal power calculation took place, sample size was smaller than the target of 100 participants. It is unknown whether the significantly higher number of AESI or death in arm 3 vs arm 1 demonstrated within the secondary analysis reflects a true safety risk of the regimen containing aspirin or is due to chance given the smaller sample size. Second, the majority participants recruited had mild TBM. The reasons for this are multiple including patients dying prior to screening (because 5 days of TB treatment was allowed prior to enrollment) and patients with decreased consciousness arriving at the hospital alone without available next of kin available for proxy consent. In the latter case, a protocol amendment allowed deferred consent for these patients, which is likely to explain in part the higher rate of mild disease in our cohort. The mild disease within our patient cohort likely explains the low level of mortality; 16% 2-month mortality contrasted with the often-quoted 50% mortality in the literature [1]. The primary end point of AESI or death was designed with the assumption that observed mortality would be near or approaching 50%. The relatively few deaths led to a greater proportion of AESI in the composite end point of AESI or death. Given that all listed AESI were proportionally more likely to occur in experimental arm 3, it is unsurprising that the number of events within the composite end point of AESI or death occurred in arm 3 where the greatest number of interventions was given. This is supported by the observation that when considering AESI alone, the cumulative incidence of events was significantly greater in arm 3, suggesting that the composite end point of AESI or death was driven by the higher rate of AESI in arm 3. The bias toward milder disease may also have affected the efficacy analysis. Given the nature of TBM and clinical trial research, it is challenging to ensure inclusion of those with severe TBM, in particular, around gaining proxy or deferred consent in unconscious patients, and ensuring early referrals to include those who are most likely to die early within the disease course. Future trials, especially phase 3 RCTs, must endeavor to overcome these hurdles and include such patients to ensure generalizability of results.

Our study, the first RCT to evaluate linezolid in TBM, demonstrates that this important drug can be safely added to standard of care to treat HIV-associated TBM. It is also the first study to date to systematically evaluate the safety of a novel drug regimen that contains enhanced antitubercular treatments alongside a host-directed therapy in TBM, demonstrating that this approach can be safe. Our results show that high-dose rifampicin and linezolid may be safely combined in HIV-associated TBM and support evaluation of the efficacy of these drugs either alone or in combination in phase 3 trials. Within our study, we did not see any significant bleeding events with the use of high-dose aspirin. A larger study is now required to see if potential harm is offset by a morbidity and mortality benefit.

## Supplementary Data

Supplementary materials are available at *Clinical Infectious Diseases* online. Consisting of data provided by the authors to benefit the reader, the posted materials are not copyedited and are the sole responsibility of the authors, so questions or comments should be addressed to the corresponding author.

## Supplementary Material

ciac932_Supplementary_Data

## Notes

***Acknowledgments.*** The authors thank the participants and their caregivers for agreeing to take part in the study. They acknowledge staff at the referring hospitals, at Western Cape and Eastern Cape provincial governments, and at the University of Cape Town Clinical Research Centre for their help in making the study a success. They thank the following Data Safety and Management Board members for their advice and guidance throughout the trial: Professor David Lalloo (chair, Liverpool School for Tropical Medicine), Dr David Meya (College of Health Sciences, Makerere University and Division of Infectious Diseases and International Medicine, University of Minnesota), Dr Evelyne Kestelyn (Clinical Trials Unit, Centre for Tropical Medicine and Global Health, University of Oxford), Dr Maryline Bonnet (Institute of Research for Development, France), and Dr Angela Crook (Medical Research Council Clinical Trials Unit, University College London).

***Disclaimer.*** For the purposes of open access, the authors have applied a CC-BY public copyright license to any author accepted manuscript arising from this submission.

***Financial support***. A. G. D. is supported by a University College London Wellcome PhD Programme for Clinicians fellowship (award 175479). S. W. is supported by the European and Developing Countries Clinical Trials Partnership (EDCTP) (grant CDF1018), Wellcome (grants 203135/Z/16/Z and 104803), and the National Institutes of Health (NIH; K43TW011421). R. P. J. L. is supported by a MRC CDA fellowship (MRC/R008922/1). R. J. W. receives support from Wellcome (104803, 203135, FC0010218), the Francis Crick Institute (which is funded by UK Research and Innovation; FC0010218), CRUK (FC0010218), Meningitis Now and NIH (R01AI45436), and EDCTP (grant to institution). G. r. M. is supported by the Wellcome Trust (214321/Z/18/Z) and the South African Research Chairs Initiative of the Department of Science and Technology and National Research Foundation of South Africa (grant 64787).
