# Supplementary material for: A Phase 2A Trial of the Safety and Tolerability of Increased Dose Rifampicin and Adjunctive Linezolid, With or Without Aspirin, for Human Immunodeficiency Virus–Associated Tuberculous Meningitis: The LASER-TBM Trial
Source: Clin Infect Dis. 2022 Dec 9;76(8):1412–22. doi: 10.1093/cid/ciac932 (PMC10110270; doi:10.1093/cid/ciac932)
Supplement: ciac932_Supplementary_Data [file ciac932_supplementary_data.docx]

**SUPPLEMENTARY MATERIAL**

**Figure S1: Study schedule**

Study design schematic describing randomisation to study arms, treatment intervention per am, visit schedule, overview of clinical procedures and timepoints relating to primary and secondary endpoint data collection

Abbreviations: RHZE: Rifampicin, Isoniazid, Pyrazinamide, Ethambutol; R_10_: Rifampicin 10mg/kg/day; R_35_: Rifampicin 35mg/kg/day; LZD: Linezolid; ASA; Aspirin;

**Table S1: Reasons for screening exclusion.**

| **Reason*** | **n** | **%** |
| --- | --- | --- |
| Chronic aspirin or NSAID use | 9 | 13.2 |
| HIV uninfected | 8 | 11.8 |
| Investigator discretion | 8 | 11.8 |
| Received more than 5 days of anti-TB treatment in 30 days prior to enrolment | 7 | 10.3 |
| Not ‘possible’, ‘probable’ or ‘definite’ TBM | 6 | 8.8 |
| Haemoglobin < 8 g/dL | 5 | 7.4 |
| INR > 1.4 | 4 | 5.9 |
| No consent for enrolment given by the patient | 3 | 4.4 |
| Peripheral neuropathy scoring Grade 3 or above on modified BPNS | 3 | 4.4 |
| Standard TB treatment contraindicated | 2 | 2.9 |
| Known rifampicin resistance during this episode | 2 | 2.9 |
| Previous drug resistant TB | 2 | 2.9 |
| Uses a 'disallowed medication' that cannot safely be stopped | 2 | 2.9 |
| Pregnant (>17 weeks at baseline) | 1 | 1.5 |
| Allergy to RHZE, LZD, aspirin | 1 | 1.5 |
| Died before enrolment | 1 | 1.5 |
| Evidence of bacterial or cryptococcal meningitis | 1 | 1.5 |
| eGFR < 20 | 1 | 1.5 |
| Platelet count < 50 109/L | 1 | 1.5 |
| Relocation prior to enrolment | 1 | 1.5 |

Abbreviations: NSAID, non-steroidal anti-inflammatory drug; BPNS; brief peripheral neuropathy score; Rif, rifampicin; RHZE; rifafour; LZD, linezolid; ASA, aspirin

*more than one reason can be assign to each participant

**Table S2: Reason for study withdrawals prior to day 56**

| **Withdrawal** | **Reason** |
| --- | --- |
| 1 | Participant relocated to Malawi and therefore unable to attend study follow up visits |
| 2 | Participant relocated to Kwazulu-Natal and therefore unable to attend study follow up visits |
| 3 | Participant withdrew consent |
| 4 | Participant withdrew consent |
| 5 | Participant developed acute psychosis and was unsafe to follow up |
| 6 | Participant lost to follow up |

**Box S1: Eligibility Criteria**

**Inclusion criteria**

- Age >18 years
- proven HIV-1 seropositivity
- Diagnosis of ‘possible’, ‘probable’ or ‘definite’ TBM

**Exclusion criteria**

- Rifampicin-resistant *M.tb* detected on any clinical specimen;
- History of allergy or hypersensitivity to RIF, isoniazid, ethambutol, pyrazinamide, LZD or ASA;
- Received more than 5 days of antitubercular therapy in the 30 days prior to screening;
- Receipt of regular daily ASA or NSAID prior to TBM diagnosis
- CSF unobtainable by lumbar puncture or another procedure;
- Evidence of bacterial or cryptococcal meningitis;
- Severe concurrent uncontrolled opportunistic infection including, but not limited to, active cytomegalovirus-associated disease, Kaposi sarcoma, *Pneumocystis jirovecii* pneumonia, HIV related or unrelated malignancy, or gastrointestinal bleeding;
- Any other form of immunosuppressive therapy, including antineoplastic and biologic agents, apart from corticosteroids;
- More than 17 weeks pregnant at baseline;
- Peripheral neuropathy scoring Grade 3 or above on the BPNS;
- Any disease or condition in which the use of the standard anti-TB drugs (or any of their components) are contraindicated. This includes, but is not limited to, allergy to any TB drug or their components;
- The presence of one or more of the following:
- Estimated glomerular filtration rate (eGFR) < 20ml/min/1.73 m2*
- INR > 1.4 and/or clinical evidence of liver failure or decompensated cirrhosis;
- Haemoglobin < 8.0 g/dL;
- Platelets < 50 x109 /L;
- Neutrophils < 0.5 x 109 cells/L;
- Any disease or condition in which any of the medicinal products listed in the section pertaining to prohibited medication is used and cannot be safely stopped;
- Known or suspected history of drug abuse or any other reason that is, in the opinion of investigators, sufficient to compromise the safety or cooperation of the participant.

*Calculated using the Cockcroft-Gault equation; INR: International normalised ration; BPNS: Brief Peripheral Neuropathy Score; NSAID: Non Steroidal Anti Inflammatory Drug;
